# Can case management improve cancer patients quality of life? A systematic review following PRISMA

**DOI:** 10.1097/MD.0000000000022448

**Published:** 2020-10-02

**Authors:** Ya-nan Yin, Yun Wang, Ni-jie Jiang, De-rong Long

**Affiliations:** a Department of Gynecology and Obstetrics Nursing, West China Second University Hospital, Sichuan University; b Key Laboratory of Birth Defects and Related Diseases of Women and Children Sichuan University, Ministry of Education; c West China Nursing School, Sichuan University, Chengdu, Sichuan, China.

**Keywords:** cancer patients, case management, quality of life, systematic review

## Abstract

**Background:**

Cancer patients are associated with a series of long lasting and stressful treatments and experiencing, and case management (CM) has been widely used and developed with the aim to increase the quality of treatments and improve the patient care services. The purpose of this review is to identify and synthesize the evidence of randomized controlled trial studies to prove that case management could be one way to address the quality of life of cancer patients.

**Methods:**

We performed a literature search in 4 electronic bibliographic databases and snowball searches were performed to ensure a complete collection. Two review authors independently extracted and analyzed data. A data extraction form was used to collect the characteristics of case management intervention, report outcomes, and quality assessment.

**Results:**

Our searches identified 3080 articles, of which 7 randomized controlled trials met the inclusion criteria. The intervention was varied from the target population, measurement tools, duration of intervention, and so on, and 5 studies consistently showed improvement in the intervention group compared with control groups, no significant difference was found between health care costs of case management care services and the routine care services.

**Conclusion:**

There is some evidence that case management can be effective in cancer patients quality of life. However, due to the heterogeneity in the target population, measurement tools, and results applied, no conclusion can be made from a meta-analysis on the present bias. More rigorously multi-centered randomized controlled studies should be provided with detailed information about intervention in future research.

## 1 Introduction

Cancer is responsible for an estimated 9.6 million deaths in 2018 and is the second leading cause of death globally.^[1]^ In recent years, developments in cancer therapy have increased the life expectancy of patients with cancer. However, many cancer patients are frequently associated with a series of long-lasting and stressful multimodal treatments and experiencing declines in psychological, physical and social functioning, which have a significantly negative impact on the quality of life.[^2^,^3^] Moreover, the health care cost of cancer can also be a heavy burden for many patients. It is reported that among the 547 long-term survivors of cancer, 20% of which were worried about affording care, and 15% of which had financial difficulties.^[4]^

Since many cancer patients have different degrees of psychological stress, mental disorder and physical dysfunction,[^5^,^6^,^7^] case management (CM) was established to use resources effectively to increase the quality of treatments and improve patient care services. The definition of case management is “a collaborative process that provides assessment, planning, implementation, coordination, evaluation to meet the individuals and familys health service needs”,^[8]^ and the case management system is carried out by substance of multidisciplinary, patient-centered and organizational care.^[9]^ Scherz et al^[10]^ concluded that case management has the potential to improve cancer patients quality of life and ease re-entry to normal life. However, in Wulff et als^[11]^ randomized controlled trial on the effects of case management in the care of colorectal cancer patients, it was found that there was no evidence that case management influenced colorectal cancer patients health-related quality of life. Therefore, the effectiveness of case management on cancer patients quality of life is not sure. Recently, a systematic review (9 experimental studies: 3 randomized controlled trials and 6 controlled before-and-after study) concluded that participants saw significant improvement in the quality of life measures with case management.^[12]^ However, little robust evidence from randomized controlled studies was available to confirm this conclusion.

The purpose of our study is to identify and synthesize the evidence of randomized controlled trial studies to prove that case management could be one way to address the quality of life of cancer patients.

## 2 Methods

### 2.1 Search strategy and study selection

We performed literature searches via 4 electronic bibliographic databases — Cochrane Library, EBSCO, ISI Web of Knowledge and PubMed from 1990 to 2018. Since all analyses were based on previously published articles, so ethical approval and patient consent were not necessary. Different combinations of words and MeSH terms were used: (“case management” OR “case manager” OR “advanced practice nurse” OR “advanced practice nursing”) AND (“cancer” OR “neoplasms”) AND (“quality of life” OR “QoL”). Besides, snowball searches were carried out to ensure a complete collection. The initial search was taken in March 2018 and was updated in April 2018. Clinical studies were included if they fulfilled the following inclusion criteria:

1. Participants are adults (>18 years) with all types of cancer or highly-probable diagnosis of cancer;
2. The only intervention is case management;
3. The outcomes should include the data of changes in quality of life;
4. Randomized controlled studies.

The case management-like interventions which fulfilled all of the following standards were included in the review:

1. The intervention includes the coordination or multidisciplinary collaboration;
2. The intervention includes in-person meeting or telephone contacting with patients;
3. The purpose of the intervention was to provide long term supports, education and information to the patients.

### 2.2 Data extraction and management

All studies were imported to End Note X7 and the duplicates were removed. Data extraction was performed by 2 review authors who analyzed and selected independently. We designed a form for included studies data extraction to attain the characteristics of case management intervention, report outcomes, and quality assessment. A third person would be consulted if differences were existing between reviewers.

### 2.3 Quality assessment

Elements from the Cochrane handbook were used to assess the methodological quality of the trials,^[13]^ which included the external and internal validity of the studies as follows: random sequence generation, blinding, allocation concealment, selective reporting, and incomplete outcome data.

## 3 Results

**Figure 1:**
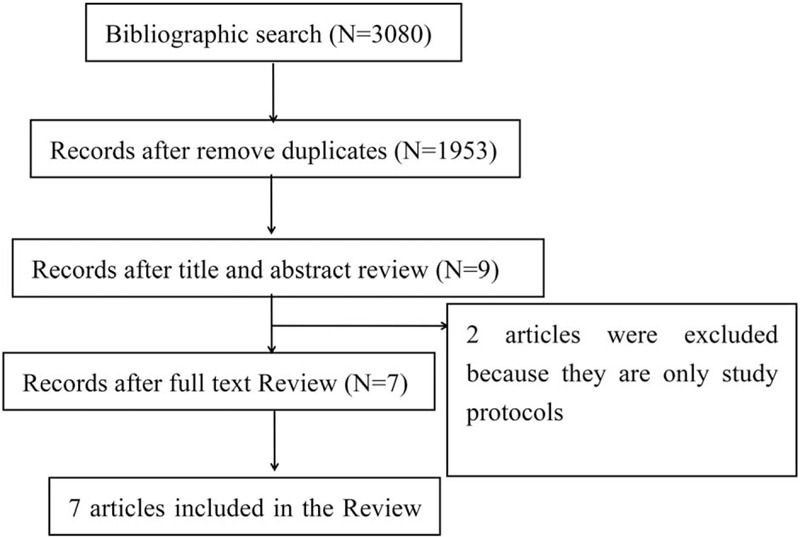
Flow chart of screening and selection process.

Figure 1 presents the flowchart of the clinical studies review process. A total of 3080 articles were retrieved and identified. After we removed the duplicates, the remaining 1953 studies were screened by title and abstract and 1944 of them were excluded, 9 articles were then evaluated by full text, of which 7 articles were included in the final review: 1 in Denmark,^[11]^ 3 in the United States of America,[^14^,^15^,^16^] 1 in Switzerland,^[10]^ 1 in the United Kingdom,^[17]^ and the only trial performed in developing countries was in Turkey.^[18]^ Of the excluded articles, both of the studies were protocols.[^19^,^20^]

Due to the heterogeneity and scarcity of outcomes, we could not conduct a meta-analysis. Therefore, we conducted a systematic review to synthesize the outcomes of all included studies. Cancer types of patients, case management intervention, contact modes, duration of intervention and so on in the 7 included studies are outlined in Table 1. The study purposes, outcome measures (tools), statistical methods and main results are reported in Table 2. Table 3 presents the quality assessment of included trials.

**Table 1:**
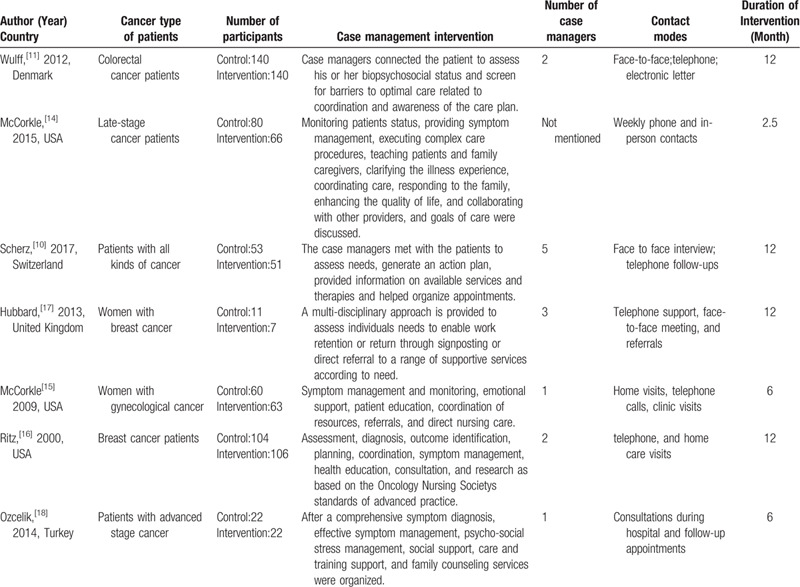
Characteristics of case management intervention in the 9 included studies.

**Table 2:**
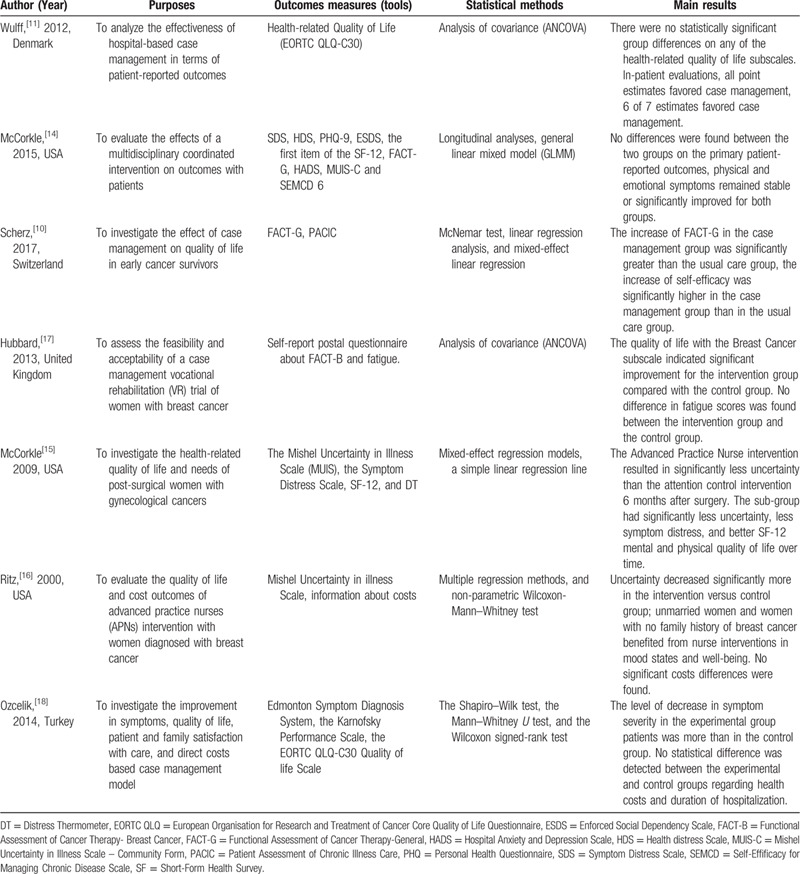
Reported outcomes of case management interventions among included studies.

**Table 3:**
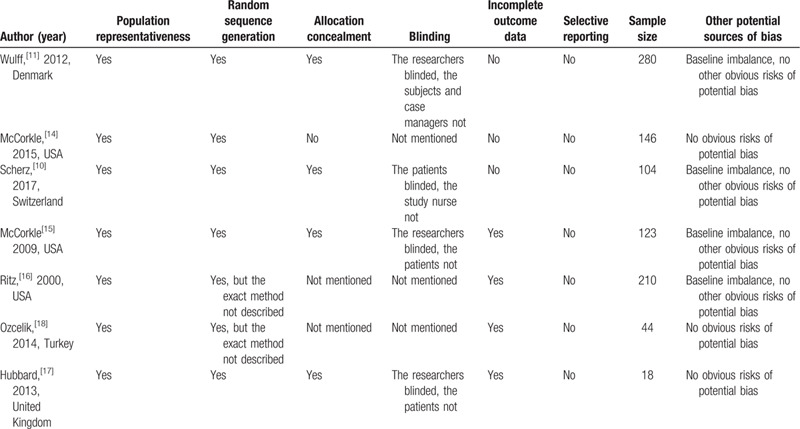
Assessment quality of included trials: randomized controlled trials.

Table 1 presents the intervention characteristics of the 7 included articles. Two studies[^16^,^17^] included breast cancer patients only, one^[11]^ studies included colorectal cancer patients only, the last 4 studies[^10^,^14^,^15^,^18^] included different kinds of cancer patients, especially advanced-stage cancer patients.[^14^,^18^] Depending on the definition of case management, all included studies fulfilled the reviewers inclusion criteria: using care co-ordination or multidisciplinary collaboration to improve the physical or psychological health status of cancer patients. Contact modes included face to face interviews, telephone follow-ups according to need, home visits, clinic visits, and referrals. Intervention duration of the 4 studies lasted for 12 months,[^10^,^11^,^16^,^17^] the rest 3 studies were respectively 2.5 months,^[14]^ 6 months^[15]^ and 6 months.^[18]^ Based on the number of cancer patients recruited in the study, the number of case managers has differed from 1 to 5.[^10^,^11^,^15^,^16^,^17^,^18^] Only one article did not mention the number of case managers.^[14]^

Table 2 audits the outcomes of all retrieved studies. Since there is no overlap of outcome measurement tools between included studies, all the articles above could not be synthesized. To further evaluate the effectiveness of case management, we categorize the outcomes into 2 domains: Quality of life and health care costs. The specific results for each study are as follows:

**Case management is effective in improving quality of life:** To investigate the quality of life for cancer patients, 7 included studies using different related scales. Both Wulff^[11]^ and Ozcelik^[18]^ evaluated the impact of case management on patients quality of life with European Organisation for Research and Treatment of Cancer Core (EORTC-C30), which was developed by Aaronson^[21]^ et al to assess the quality of life of patients who diagnosed with cancer. EORTC-C30 has 4 domains: physical, emotional, cognitive and social functions, and a higher score indicates better functioning. The Functional Assessment of Cancer Therapy - Breast Cancer (FACT-B)^[22]^ was used to evaluate breast cancer-related quality of life. Hubbard^[17]^ and Ritz^[16]^ measured the health-related quality of life with the FACT-B among breast cancer patients. FACT-G^[23]^ was used for general cancer patients assessment. Scherz^[10]^ and McCorkle^[14]^ sent out the FACT-G questionnaire to measure the quality of life in all kinds of cancer patients. The Short-Form Health Survey (SF-12)^[24]^ consists of 12 items and represents 2 components: physical and mental health, and it was used in 2 studies.[^14^,^15^] Other related scales included: Symptom Distress Scale (SDS); Health distress Scale (HDS);

Personal Health Questionnaire (PHQ); Enforced Social Dependency Scale (ESDS), Hospital Anxiety and Depression Scale (HADS); Mishel Uncertainty in Illness Scale – Community Form (MUIS-C) and so on.

When categorizing outcomes and taking nothing else into account, 5 articles proved that case management can be effective to improve the quality of life of cancer patients in different dimensions.[^10^,^15^,^16^,^17^,^18^]

**No significant difference was found between health care costs of case management care services and the routine care services:** Two studies[^16^,^18^] reported the health cost outcomes based on case management intervention. Rits^[16]^ evaluated the cost outcomes of intervention with women newly diagnosed with breast cancer, and the results showed that there are no significant differences between the 2 groups in either reimbursements or overall charges. These results were consistent with Ozceliks^[18]^ study.

### 3.1 Methodological quality

**Figure 2:**
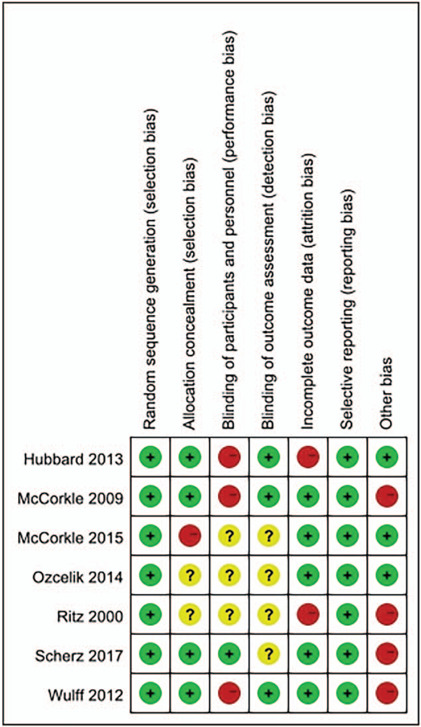
“Risk of bias” summary: review authors judgements about each risk of bias item for each included study.

Overall, the 7 included studies had a moderate risk of bias (Fig. 2). Table 3 was the methodological quality assessment of the included studies. All of the 7 included trials claimed that randomization was performed during the intervention. However, 2 articles[^16^,^18^] did not describe by the exact process of random sequence generation. McCorkle^[14]^ randomized the head and gastrointestinal clinics to the routine care group, lung and gynecologic clinics to the intervention group, so there was a high risk of allocation concealment. The information on allocation concealment was not mentioned in 2 trials.[^16^,^18^] Only one trial^[10]^ of the 7 studies reported that the participants were blinded to group allocation, while the others not mentioned. Four articles[^10^,^14^,^16^,^18^] did not report the details about blinding to researchers. In Ritz,^[16]^ 58 participants were excluded from the health cost analyses because of missing substantial amounts of data. Hubbard^[17]^ recruited 23 participants, but only 18 women were finally been analyzed. The obvious risk of selective reporting was not found in the included articles. In Ritz,^[16]^ the baseline was imbalanced between 2 groups in hormone therapy and histology. In Wulff,^[11]^ baseline imbalance was observed in health-related quality of life score (except for cognitive functioning). In McCorkle,^[15]^ 2 groups were not balanced at baseline on quality of life measures. In Scherz,^[10]^ baseline imbalances were observed in the patient-reported FACT-G score. No obvious risk of potential sources of bias was found in other trials.

## 4 Discussion and implications

### 4.1 Principal findings

We included 7 randomized controlled trials that evaluated the effectiveness of case management on cancer patients quality of life in this systematic review. Five articles consistently showed improvement in the quality of life among cancer patients compared with the control group. But the target group, measurement tools, and results were heterogeneous, so it was hardly possible to conduct a meta-analysis of the results.

As a result, there is some evidence that case management can be effective in cancer patients quality of life, but no conclusion can be made from a meta-analysis on the present bias.

## 5 Discussion and implications

The principal strength of this review was all the included articles were randomized controlled studies, though 2 of them did not tell the exact method of random sequence generation and allocation concealment. Another strength is our wide search strategy, a total of 3080 articles were retrieved and identified.

In recent years, there has been a tendency toward the use of case management as a method to improve the continuity of care for patients with cancer.^[25]^ Investigators have proved that case management can be effective in the quality of life and psychological well-being of the patients.[^9^,^26^] Whereas, others reported no significant improvement in the quality of life outcomes.[^27^,^28^] Wulff^[25]^ believed that the reason for different results may be the weak definition of case management. Since case management is a clinical behavioral intervention, and tailored, specialized care was offered directly or indirectly by individual case managers, it is hard to conclude which aspect(s) contribute to the overall effect,^[29]^ so case management is also regarded as a “black box”.^[30]^ However, despite case management models diverge from their designs, intervention methods and outcome measures. The intervention contains essential components: similar definitions and principles.^[25]^ According to the intervention model of case management, case managers offered continuity of health care services when medical follow up appointments were less frequent or ceased.^[31]^ Researches in our review showed that cancer patients would acquire better physical and psychological status through symptom management, assessment of needs, direct referrals, and other services provided by the case managers.[^16^,^17^,^18^,^19^,^20^] Therefore, to get effective outcomes, it is important to carry out correct dosage of services, which means the intervention contents, numbers of case managers, contacts modes, quantities of intervention and the intervention duration must be monitored and recorded, and case managers must be trained with ability and skill to follow the intervention protocols.^[32]^

Quality of life includes physical, role, emotional, cognitive and social functions, and it may be affected by many different factors, such as an individuals beliefs, experience, perceptions, and expectations.^[18]^ Since all of these results were self-reported by cancer patients, they may be overestimated and can not represent the real difference between the 2 groups.^[5]^ Information bias can not be avoided because all the participants were not blinded and they have been informed about the aim of the study,^[10]^ the control group may receive a higher quality treatment than routine care.^[33]^ To reduce information bias, neutrally informed the purpose of the research at recruitment is needed.^[11]^

Since 7 included studies reported quality of life measures with different measurement tools, the outcomes of improvement in the intervention group compared with routine care groups were not universal. Thus, to avoid the heterogeneous outcomes, it is essential to develop more measurements with high reliability and validity. Four trials in our review targeted diverse kinds of cancer patients, to know the effect of case management on certain type precisely, more researches need to be conducted to target certain types of cancer.

One of the fundamental purposes of case management is to reduce health care costs. It was reported that the health care costs of cancer patients in an interdisciplinary palliative care study fell by between US$14,486 and US$21,252.^[34]^ However, in our review, we found no significant difference between health care costs of case management care services and routine care services. Thus, more trials are needed in order to investigate the effect of case management on health care costs.

Publication bias may have reduced the number of articles found, but it was always a problem when performing a systematic review.

### 5.1 Limitations of this review

The main limitation of this systematic review were the weak definition of case management and the diverse outcomes, our search strategy may also blur the boundary of include and exclude criteria. This review only included researches published in English, however, more evidence may be found published in other languages. Never the less, since the articles included were conducted in 5 different countries and the health care systems may be also different, which may influence the effect of case management.

## 6 Conclusion

In summary, due to the heterogeneous outcomes, this systematic review suggested that no reliable conclusion can be made about the effectiveness of case management on cancer patients quality of life. Thus, to open the “black box”, more rigorously multi-centered randomized controlled studies should be provided with detailed information about intervention in future research.

## Author contributions

Ya-nan Yin was contributed to the research design, implementation and was the principal author of the manuscript. Yun Wang and Ni-jie Jiang participated in the literature review and data collection and contributed to the first draft of the manuscript. De-rong Long was involved in the consultation and contributed to the final draft of the manuscript.

**Methodology:** De-rong Long.

**Project administration:** Ya-nan Yin, Yun Wang, Ni-jie Jiang.

**Supervision:** Ya-nan Yin, De-rong Long.

**Writing – original draft:** Ya-nan Yin, Yun Wang.

**Writing – review & editing:** Ni-jie Jiang.
